# Reproduction and metamorphosis in the *Myristica* Swamp tree frog, *Mercurana myristicapalustris* (Anura: Rhacophoridae)

**DOI:** 10.7717/peerj.5934

**Published:** 2018-11-21

**Authors:** Robin Kurian Abraham, Jobin Kuruvilla Mathew, David Valiaparampil Raju, Ramprasad Rao, Anil Zachariah

**Affiliations:** 1 Biodiversity Institute and Department of Ecology and Evolutionary Biology, University of Kansas, Lawrence, KS, USA; 2 Karakkattupeedicayil, Edakkara, Malappuram, Kerala, India; 3 Valiyaparampil House, Kuzhimattom, Kottayam, Kerala, India; 4 Chathurthi, Mudradi, Udupi, Karnataka, India; 5 Beagle, Chandakunnu, Wayanad, Kerala, India

**Keywords:** India, Amphibia, Rhacophoridae, Reproductive mode, *Myristica* swamp

## Abstract

The reproductive biology of the *Myristica* Swamp tree frog (*Mercurana myristicapalustris*), a monotypic rhacophorid frog endemic to the foothills of the Western Ghats mountains of India, has remained unknown since the description of the genus and species. We monitored individuals from parental generation amplexus to the completion of offspring generation tadpole metamorphosis. Surprisingly, our observations revealed that this species exhibits many previously unknown characteristics, including the first ever record of the female, and a diverse call repertoire, consisting of five different call types (the functions of which remain incompletely known). We were also able to determine that reproductive activity peaked during the late pre-monsoon season, that males engaged in intraspecific aggressive encounters to occupy and to defend desirable territories, and that oviposition took place in terrestrial nests made by females. Embryonic development in the unattended nest was followed by tadpole development, which concluded within 40 days. The specific breeding mode employed by *Mercurana*, which restricts its range to the endangered *Myristica* swamp ecosystem, likely renders it susceptible to multiple threats, which should be considered jointly in future conservation planning.

## Introduction

Anuran reproduction has conventionally been classified into two broad suites of breeding systems: explosive and prolonged, even though they are opposite extremes of a broad spectrum (Wells, 2007). Explosive breeding is usually associated with habitats in seasonal climates, in species that breed only once each year, in aggregations around suitable oviposition sites (Wells, 2007). In many species that breed explosively, males congregate for only a few days of the year, form ‘choirs’ and vocalize in choruses, advertising locations of breeding sites to females that arrive simultaneously from surrounding areas (Taylor, 1977; Wells, 1977); in such species, with the arrival of females, the males stop singing and start competing energetically and pursue females to ensure mating opportunity (Duellman & Trueb, 1986). In prolonged breeding, on the other hand, breeding can last many months and males establish individual territories and use different calls both to warn male rivals not to approach their territory and to stand out from the rest of the males and therefore be chosen by females (Gerhardt, 1991; Wells, 1977). Between these two general classes, around 60 reproductive modes have been identified in anurans, ranging from aquatic eggs deposited in water to vivparity (Crump, 2015; Haddad & Prado, 2005; Wells, 2007).

The Asian tree frogs (family Rhacophoridae) comprise 421 recognized species (Frost, 2018), which exhibit four general reproductive modes: aquatic breeding, terrestrial gel nesting, terrestrial foam nesting, and direct development (Meegaskumbura et al., 2015). In 2013, we described two new monotypic rhacophorid genera from the southern Western Ghats mountains of India (Abraham et al., 2013). Of these, *Mercurana* is a lineage that is sister to the clade containing direct-developing *Raorchestes* + *Pseudophilautus*. Other, early, successively-branching taxa include *Nasutixalus* (three species*,* from Northeast India, Myanmar, and Southern Tibet and Yunnan in China (Yang & Chan, 2018), *Kurixalus* (15 species distributed from the Eastern Himalayas to insular Southeast Asia (Lv et al., 2018), and *Beddomixalus* (one species, from the southern Western Ghats (Abraham et al., 2013). All six genera are part of a larger clade including direct-developing taxa such as *Philautus* (55 species, primarily insular Southeast Asia), *Raorchestes* (62 species, primarily in the Indian Subcontinent), and *Pseudophilautus* (79 species, primarily in Sri Lanka) (Frost, 2018). The monophyly of this large clade traditionally has been interpreted as supported by a single unambiguous synapomorphy: the presence of a simple, tubular Wolffian duct in males (Abraham et al., 2013; Liem, 1969). However, within this clade, the phylogenetic position of *Mercurana* is not entirely clear with respect to the clades of *Raorchestes* and *Pseudophilautus* (Biju et al., 2016; Meegaskumbura et al., 2015).

Previously, we documented the reproductive behaviour of *Beddomixalus bijui* in detail: the species breeds explosively, ovipositing terrestrial, non-pigmented eggs openly on the moist bed of highland swamps immediately following the first pre-monsoon rains (Abraham et al., 2013). Embryos develop into free-swimming tadpoles that complete larval growth in swamps that inundate with standing water after the rains intensify (Abraham et al., 2013). This strategy was classified as ‘terrestrial gel nesting with exotrophic tadpoles’ by Meegaskumbura et al. (2015).

The Western Ghats mountain range is a globally important biodiversity hotspot that supports many unique ecosystems and numerous endemic families, genera and species of plants, animals and fungi (Gunawardene et al., 2007). This region also represents a major area of endemic amphibian radiations, with hundreds of described species (Bossuyt et al., 2004; Vijayakumar et al., 2016). *Mercurana myristicapalustris* is a rare, highly range-restricted species that has so far been located only at two sites in the Western Ghats, both in *Myristica* swamp forests in the southern part of the state of Kerala. *Myristica* swamp forests are dominated by species of Myristicaceae (nutmeg family), and are found as pockets in low-lying, poorly-drained depressions in evergreen forest formations in the Western Ghats (Chandran & Mesta, 2001; Moorthy, 1960). Once widespread across the west coast of India, these relics of primeval forests occur today as small fragments from Goa in the north to southern Kerala in the south (Limaye, Padmalal & Kumaran, 2017). We previously noted reproductive behaviour in *M. myristicapalustris*, which appeared similar to that of *Beddomixalus* (Abraham et al., 2013), as likely indicative of explosive breeders. As in *Beddomixalus*, we observed large aggregations of male *Mercurana* congregating at breeding sites during the pre-monsoon period, vocalizing males calling from perches, and females depositing eggs on the swamp floor.

Males of some species in *Pseudophilautus*, *Polypedatus*, and *Rhacophorus* are known to defend limited calling and mating areas against conspecifics with combat, suggesting territoriality (Arak, 1983; Feng & Narins, 1991; Kasuya, Hirota & Shigehara, 1996). We witnessed one instance of physical combat between males of *M. myristicapalustris*, in what was perceived to be competition for optimal breeding sites (Abraham et al., 2013).

Oviposition by female *M. myristicapalustris* occurs in shallow burrows, excavated by the female in the loamy swamp floor (Abraham et al., 2013), suggesting that a rainfall-mediated stimulus may be vital for further larval development. The related *Kurixalus idiootocus*, from Taiwan, lays pigmented eggs in shallow depressions or holes at the edges of temporary ponds (Kuramoto & Wang, 1987); embryonic development apparently is stimulated by heavy rain, and tadpoles complete development in water, much like in *Mercurana* and *Beddomixalus*.

In this study, we use extensive new field observation and natural history data to test if *M. myristicapalustris* (a) is an obligate swamp breeder (b) employs terrestrial nesting, (c) is territorial, and (d) is an explosive breeder, typical of many other pre-monsoon breeding frogs in India.

## Methods

We monitored a population of *M. myristicapalustris* from 5 May–31 July 2016. The study area is a *Myristica* swamp forest patch, approximately 1,500 m^2^, in the 560,000 m^2^ Ammayambalam Forest (8.841°N, 77.035°E, ∼170 m elevation), on the periphery of the Kulathupuzha Reserve Forest, in the state of Kerala, India. We also observed male activity (but no breeding) at Sasthanada swamp, a much smaller *Myristica* swamp patch 3.8 km southeast in aerial distance from our primary study site. Both these sites form discharge sites for the headwaters of the Pu Ar stream which is a tributary of the Kulathupuzha river, which in turn drains into the Kallada River. We assessed reproductive behaviour four or five nights/wk during the pre-monsoon (May to June) season, and larval development during the early course of the subsequent southwest monsoon (June to July) season. We located 25 males around a sandy depression that flooded during the monsoon rains, and we visited the site every evening. Adult males started making calls in the evenings from 11 May 2016, and vocalization activity persisted till 5 June 2016. We noted an increase in number of calling males with the progression of days peaking between 18–26 May, after which we sensed a decline in number of individuals calling; number of calling individuals per evening was not counted.

We recorded five advertisement calls (vocalizations) from each of four males with a TASCAM™ HDP2 portable stereo recorder, in uncompressed .wav format at a sampling rate of 44.1 kHz and 16-bit resolution. Individuals were recorded for three min at a distance of 50–200 cm, and calls were visualized using the SEEWAVE R package (Sueur, Aubin & Simonis, 2008; Team RDC, 2013) and acoustic properties were analyzed in Raven©Pro 1.5 (Bioacoustics Research Group, Cornell Lab of Ornithology 2012). All calls were recorded at an ambient temperature range of 25.5  ± 1.5 °C, and temperature around the frog was measured using a Fluke 62 MAX InfraRed Thermometer. We did not measure SVL of males recorded to minimize disturbance which might bias reproductive behaviour. Dominant frequency was measured at peak amplitude; limited sample sizes prevented statistical summaries of rate-related call characters.

We observed combat behaviour at 19:22 h on 27 May 2016, at an ambient temperature of 27 °C and 100% humidity. We photographed male–male antagonistic interactions, and male–female interactions. We noted oviposition site, located clutches, and counted eggs per clutch. We collected tadpoles with a dip net, and euthanized specimens in 5% lidocaine, before preservation in 10% neutral-buffered formalin. We staged (Gosner, 1960), photographed, and took morphological measurements (to the nearest 0.1 mm, with Mitutoyo callipers, following McDiarmid & Altig, 1999) from preserved specimens.

Measurements included total length, body length, tail length, body width, body height, maximum tail length, upper tail fin height, lower tail fin height, snout-spiracle distance, internarial distance, naris-snout distance, eye-naris distance, interorbital distance and eye diameter (Table 1).

**Table 1:** Morphometric measurements (in mm) of two tadpole specimens (Stage 38) of *Mercurana myristicapalustris*.

	**TL**	**BL**	**TAL**	**BH**	**ED**	**TMH**	**MTH**	**UTF**	**LTF**	**NSD**	**END**	**IND**	**IOD**	**SSD**	**HW**	**BW**	**TMW**
Tadpole 1	22.1	06.9	15.2	02.8	00.9	02.4	02.4	00.9	00.3	01.2	00.9	00.1	01.6	04.4	03.9	03.5	02.1
Tadpole 2	20.9	06.6	14.3	02.7	00.8	02.3	02.3	00.9	00.2	01.1	00.8	00.1	01.5	04.2	03.7	03.3	02.0

**Notes.**

TL Total length BL Body length TAL Tail length BH Body height ED Eye diameter TMH Tail muscle height MTH Maximum tail height UTF Upper tail fin height LTF Lower tail fin height NSD Nasal snout distance END Eye nasal distance IND Inter nasal distance IOD Inter orbital distance SSD Snout spiracle distance HW Head width BW Body width TMW Tail muscle width

Five tadpoles of the same clutch were captured after emergence and reared together at our field base 60 kms from the collection site, also in the foothills of the Western Ghats, until completion of metamorphosis to confirm species identification, confirm staging, and study development series and duration. Tadpole rearing was done at ambient temperatures ranging between 23–31 °C in a glass tank with water, soil substrate and associated debris collected from the natural site of the clutch. Fieldwork at the designated site was conducted with permissions and guidelines from the Kerala State Forest Department under the research permit # WL10-38972/2016. This study has been approved by the University of Kansas IACUC under protocol # AUS 158-04.

## Results

**Figure 1:**
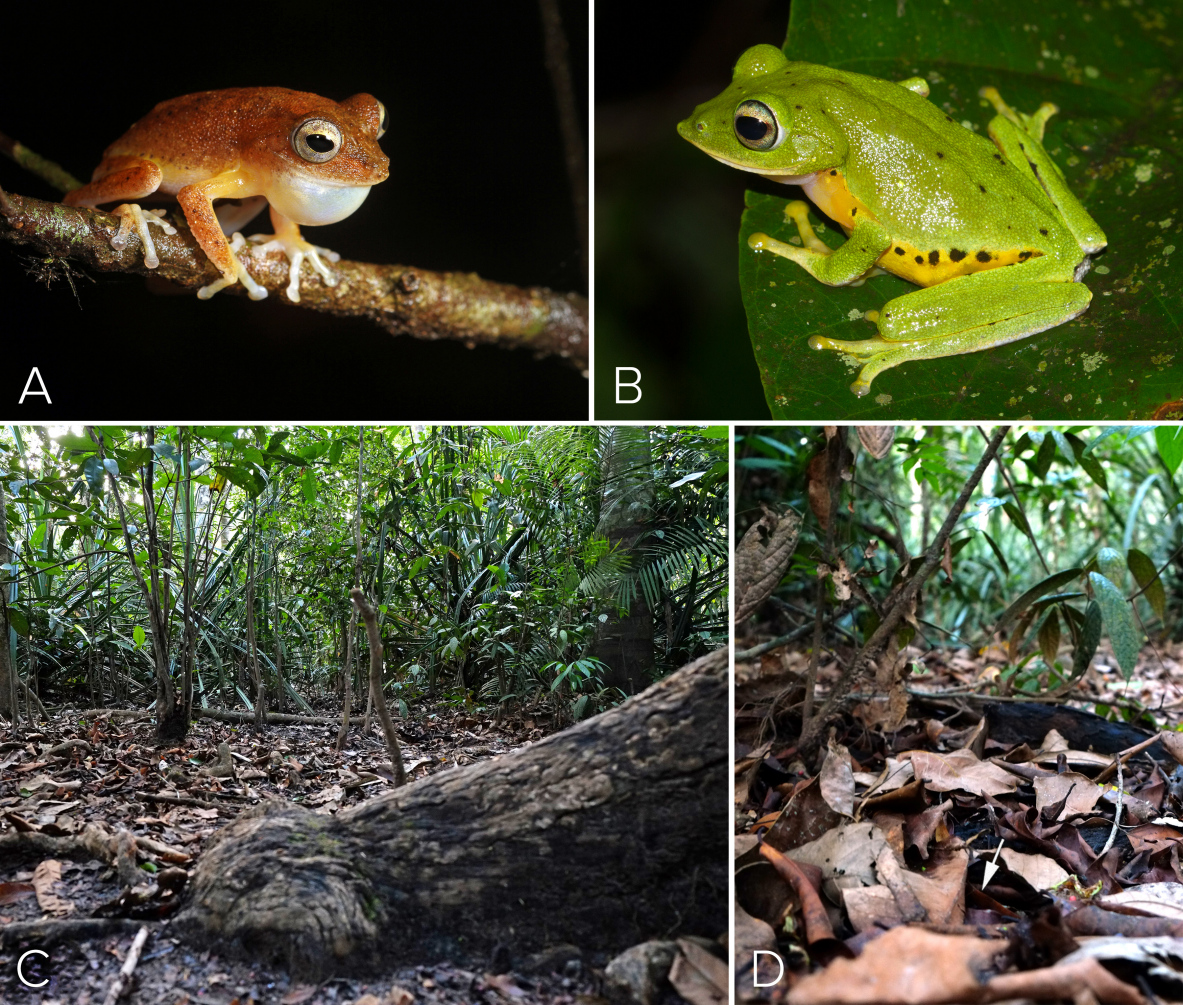
Habit and Habitat of *M. myristicapalustris*. (A) Male vocalizing from a branch perch. Image credit: David V. Raju; (B) female. Image credit: Jobin Mathew; (C) swamp forest floor before the first pre-monsoon showers. Image credit: Robin K. Abraham; (D) typical ovipostion site under leaf litter on the swamp floor beneath male perch (arrow, location of burrow). Image credit: Robin K. Abraham.

Males vocalized from perches (e.g., slender trunks of tree saplings, or branches/foliage of understorey vegetation such as *Gomphandra tetrandra*, *Myxopyrum smilacifolium*, *Phrynium pubinerve*, the screw palm *Pandanus thwaitesii*, the rattan palm *Calamus hookerianus* and the aroid *Lagenandra ovata* among a few) 0.25–1.5 m above ground (Fig. 1A), during the intervals when the intermittent rainfall subsided. Although individual males call at moderate levels of sound intensity, calls of males in the vicinity of our study area became temporally synchronized, collectively resulting in a loud chorus, which could be heard by approaching field biologists 20 m away. Synchronized choruses lasted 10–12 s, separated by 480–600 s.

**Figure 2:**
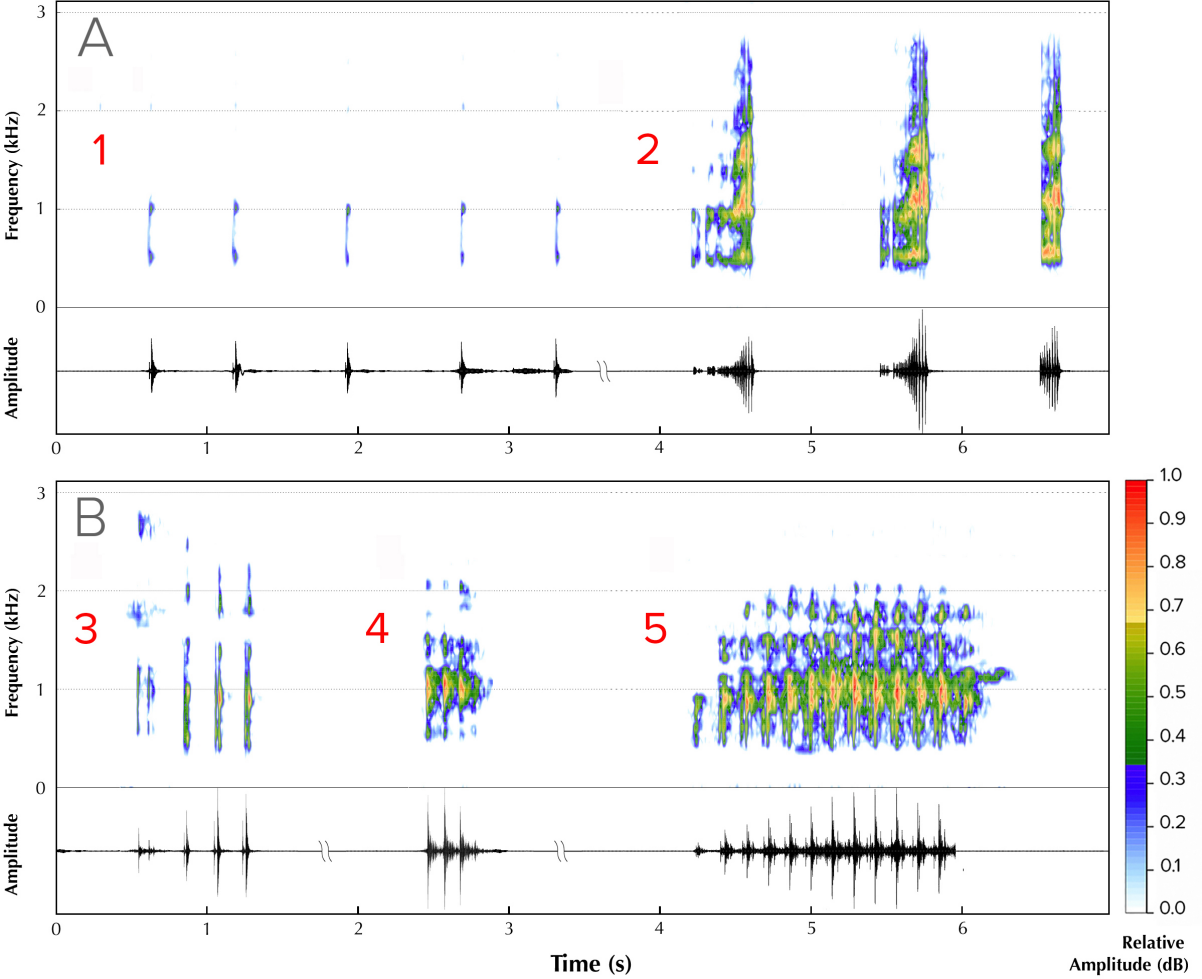
Advertisement vocalizations of *Mercurana myristicapalustris*. Comparative spectrograms and corresponding oscillograms of different call types (A, Types 1 & 2; B, Types 3, 4 and 5). Audio credit: (1, 2 & 5) Robin K. Abraham and (3 & 4) Ramprasad Rao.

Five different advertisement call types were recorded from four males (Fig. 2). Type 1 (all calls recorded between ambient temperatures of 25.5 ± 1.5 °C) consisted of a series of amplitude modulated pulses with a dominant frequency of 1,895 Hz, each separated by average interpulse intervals of 0.32 s (mean = 0.32, range = 0.26–0.36, SD = 0.30, *n* = 5; Fig. 2A, Type 1). The pulse rate was 2.94 pulses/s and the call duration was 1.76 s. Type 2 (all calls recorded between ambient temperatures of 26 ± 1 °C) consisted of screechy pulses, with a dominant frequency of 2,182 Hz (*n* = 3), separated by an interpulse interval of approximately 0.38 s. Each pulse in this call type was strongly amplitude modulated, with the amplitude peaking over the last 50% of pulse length duration (Fig. 2A, Type 2). The pulse rate was 0.57 pulses/s and the call duration was 1.24 s. We were able to record only one instance each of a third, fourth and fifth call type. Type 3 (call recorded at an ambient temperature of 25.4 °C) consisted of four pulses, of which the first one was distinct and dominant frequency was 1,808 Hz (Fig. 2B, Type 3). The interpulse interval was approximately 0.075 s, the pulse rate 0.12 and call duration 0.36 s. Type 4 (call recorded at an ambient temperature of 24.5 °C), with a call duration of 0.32 s, consisted of a rapid, three-pulse call with a dominant frequency of 2,756 Hz and no interpulse interval (Fig. 2B, Type 4). Type 5 (call recorded at an ambient temperature of 25.2 °C), with a call duration of 0.94 s, had 13 rapid pulses and had a dominant frequency of 1,895 Hz and also did not have a measurable interpulse interval (Fig. 2B, Type 5).

**Figure 3:**
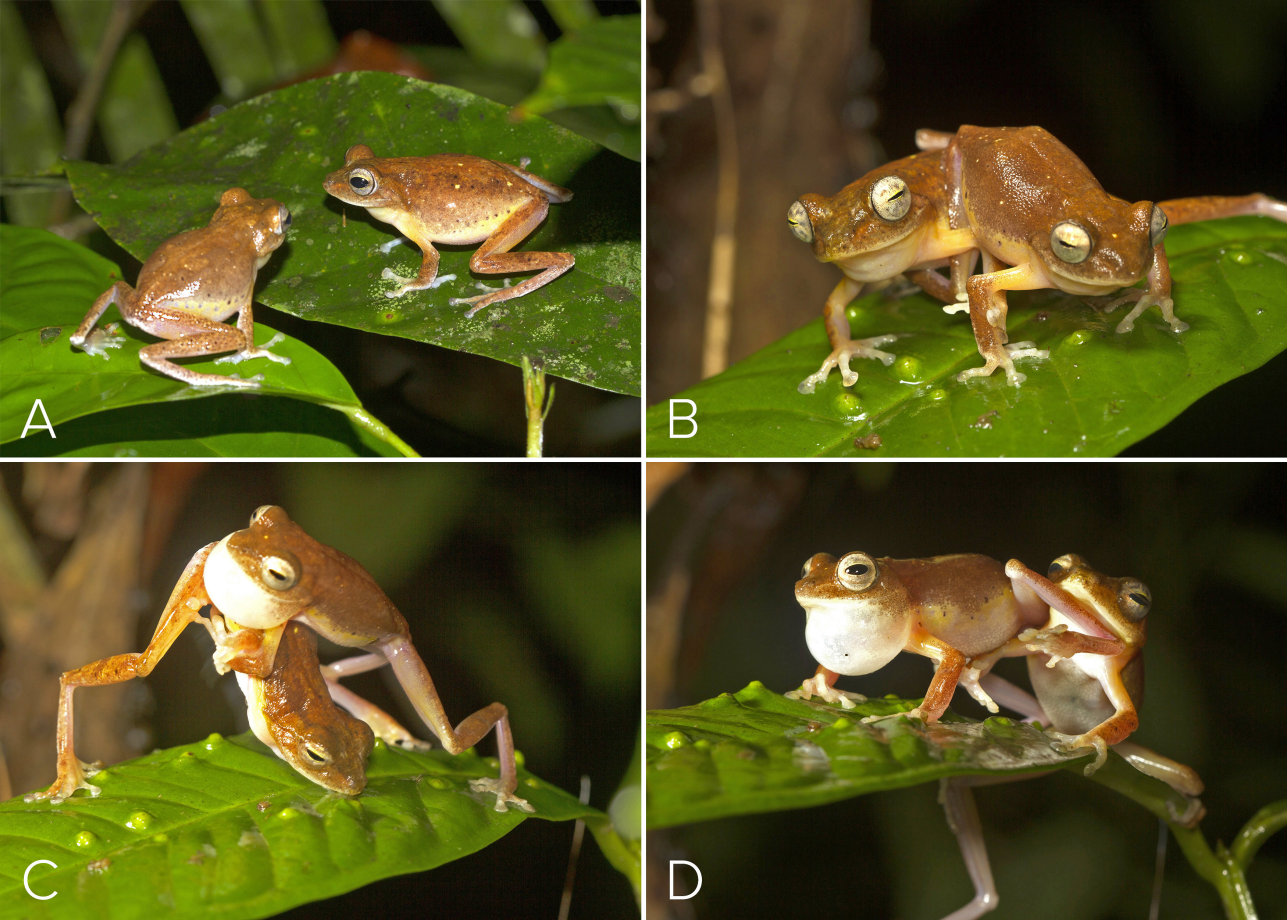
Male-male combat in *Mercurana myristicapalustris*. (A–D) Combat for perch position on a leaf. Image credit: David V. Raju.

Males occupied specific perch sites every night for our 2-wk study period. These sites were located in the parts of the swamp where the canopy was broken, making understorey vegetation growth possible. Individual males could be identified by the specific arrangement of yellow spots and blotches (in relation to smaller black speckles) on their dorsal skin surface, the pattern which is unique to each individual (Fig. 3A shows an example of two males that can be distinguished by the differences in their dorsal spot pattern). Two males ventured onto neighbouring male perches. On 27 May 2016 (19:22 h), one male approached an adjacent male at its habitual leaf perch. The resident male intensified calling rate upon detecting the intruder’s approach, and physical combat began when the intruding male arrived on the resident’s leaf (Figs. 3B–3D). Combat consisted of pushing, wrestling and kicking while calling at the same time, till one of the two individuals either withdrew or was pushed off the perch. The combating males made the Type 2 call (Fig. 2B), which comprised of screechy notes (pulses), and were louder than the other call types. After 240 s of physical combat, the intruding male retreated. A second instance of male-male combat was observed (20:08 h, 29 May 2016), involving two males violently kicking one another using their hind limbs, till one fell out of the perch.

**Figure 4:**
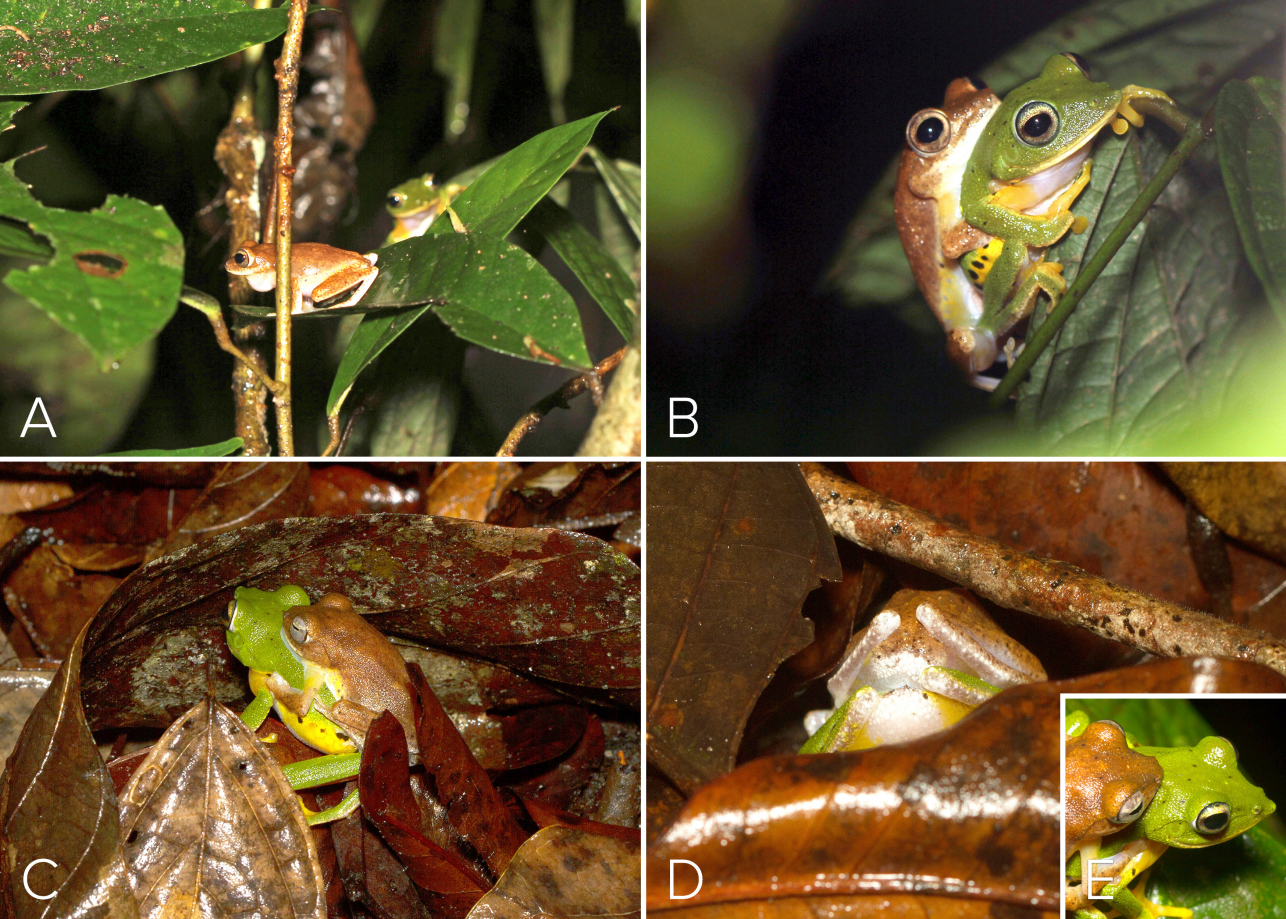
Courtship and amplexus in *Mercurana myristicapalustris*. (A) Female approaching a vocalizing male atop leaf perch; (B) male and female in amplexus; (C) amplectic pair descending to forest floor beneath perch site; (D) female digging burrow in the soil under the leaf litter to prepare oviposition site; (E) snout profile of pair highlighting pronounced snout of female which is used as an appendage to dig soil. Image credit: David V. Raju.

**Figure 5:**
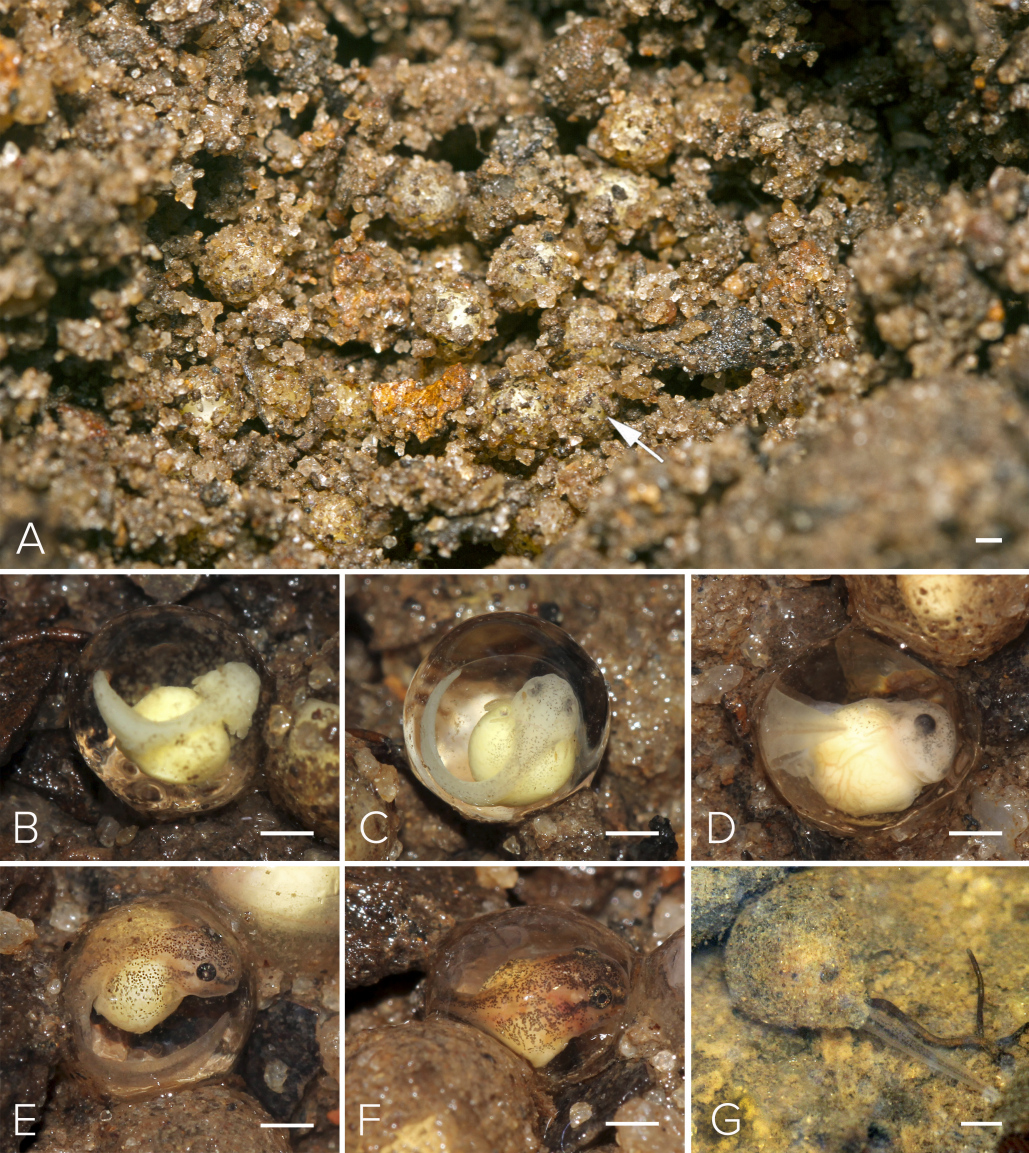
Nest and larval development of *Mercurana myristicapalustris*. (A) Egg clutch in the soil nest (arrow, freshly deposited eggs), and embryos at approximate Stages of (B) 19, (C) 20, (D) 21, (E) 22, (F) 24 and (G) 26. Bar represents 1 mm. Image credit: Jobin Mathew.

At 21:05 h on 27 May 2016, a female was observed in midstory vegetation layers as it approached a different conspecific male vocalizing on a lower perch above the swamp (Fig. 1B). The female descended to the male’s perch (Fig. 4A) and that male engaged in axillary amplexus (Fig. 4B) at 22:45 h, after which the pair descended to the forest floor and entered the leaf litter to access the soil substrate below the male’s perch plant (Figs. 4C–4D). The female (still in amplexus) used her pointed snout (Fig. 4E) to make a shallow burrow in the soil. She then turned around to position the posterior ends of the pair over the freshly made burrow, and initiated oviposition. The oviposition site was located in one of the lowest sections of the swamp, which had not yet been inundated during the pre-monsoon. A clutch of ∼130 non-pigmented eggs was deposited in this nest during a period of about 50 min (Fig. 5A). Egg size at the time of oviposition was 2.5 mm (*n* = 15). The female then used her hind legs to mix the eggs with the substrate soil (Fig. 5A), after which the amplectant pair emerged from the leaf-litter before disappearing from view. We located 3 other clutches in the vicinity (i.e., within a 10 m radius), all in the lowest depression of the swamp floor.

**Figure 6:**
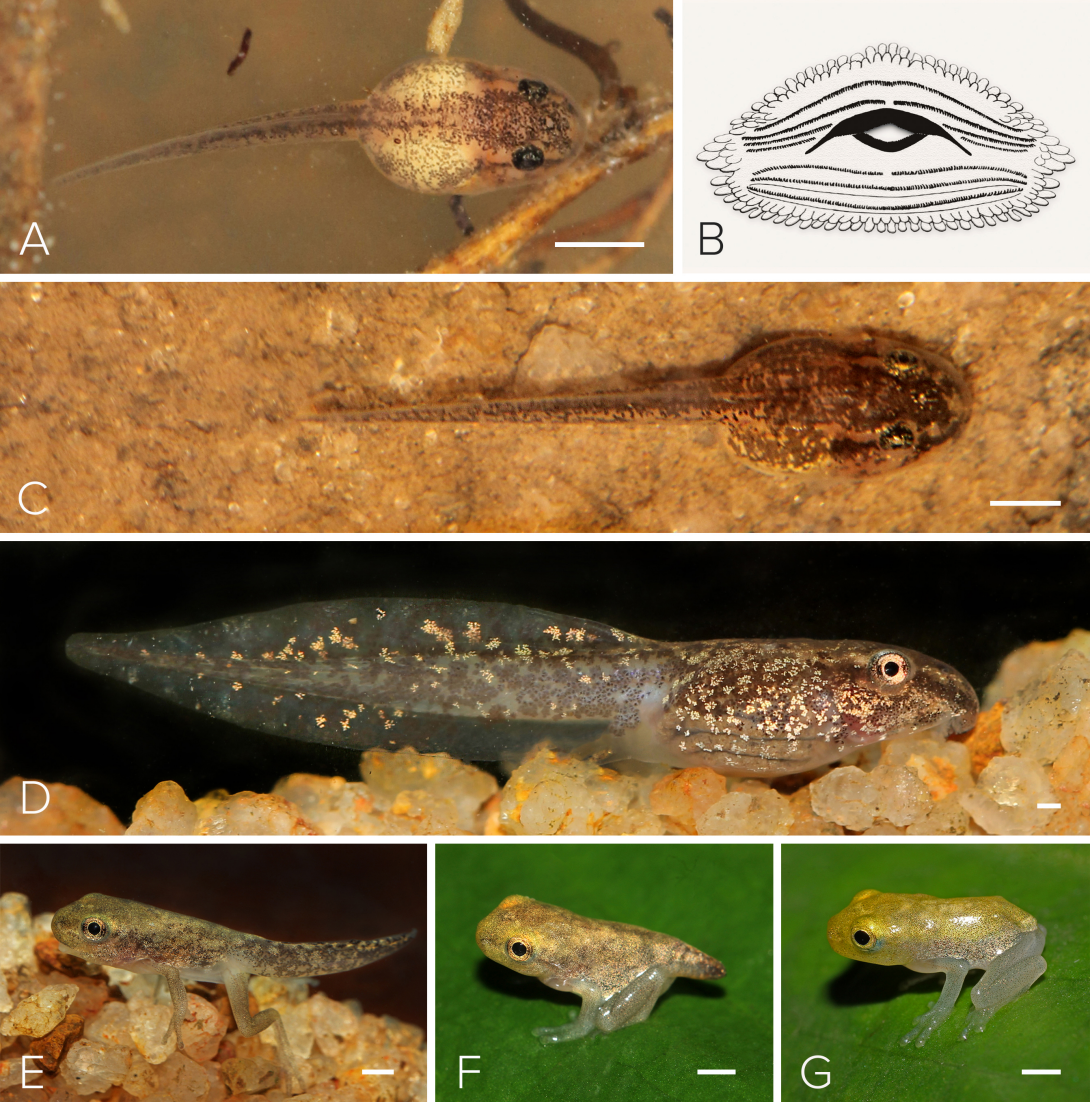
Tadpole of *Mercurana myristicapalustris.* Stages (A) 28, (B) illustration of the oral apparatus (LTRF: 4(2-4)/3(1)). Image credit: Robin K. Abraham, (C) 29, (D) 30 (E) 42 (F) 44 and (G) 45. Bar represents 1 mm. Image credit: Jobin Mathew.

Motile larvae (Stage 25) emerged from the jelly capsule after seven days of embryonic development (Figs. 5G & 6A). Our impression was that this tadpole emergence seemed to be in response to the accumulation of rainwater inundating the nest and surrounding immediate area. We observed delayed emergence after 10 days in clutches (fertilized on the same night) not submerged by flooding of the forest floor. We had observed a decline in rainfall during May–June 2016 as compared to the previous year, and this was corroborated with annual rainfall data collected from the Kerala Livestock Development Board Bull Station & Farm, Kulathupuzha, which was the nearest rain gauge we had access to (see Supplementary File & Fig. S2).

We selected a nest with a clutch of ∼120 eggs for observation in May, 2016 (Fig. 5A). We measured 10 eggs and a few embryos in the nest, for the following developmental stages. The freshly fertilized eggs had an average diameter of 2.5 mm (mean = 2.49, range = 2.3–2.6, SD = 2.36, *n* = 10). The jelly layer was not obviously visible at this initial stage. After 66 h, an embryo had grown to 3.9 mm (including a prominent jelly layer) at Stage 19 (Fig. 5B); gill filaments were present, and the tail elongated. After 80 h, at Stage 20, pigment appeared on the body and eye, and vascularization of yolk was noted (Fig. 5C). At Stage 22 (91 h), tail-fin differentiation began, pigmentation of the eye and body intensified, and the gill filament increased in size (Fig. 5E). At Stage 23 (131 h), deeper invagination of the oral groove and development of mouthparts were noted and external gills began to atrophy and pigmentation was prominent. At Stage 24 (140 h), mouthparts differentiated and movement of embryos within the jelly layer was observed (Fig. 5F). After 150 h, at Stage 25, the yolk was replaced by a coiled gut and, within a few hours, the larva emerged from its jelly sac into the pool of water that had collected in the swamp basin following heavy showers of the southwest monsoon. The larva now had a total length of 7.7 mm. After 175 h, still at Stage 26 and with a length of 11.6 mm, all labial tooth rows and jaw sheaths had keratinized (Fig. 5G). The free-swimming tadpole hid under leaf litter of the swamp pool, and fed from the loamy substrate. After 34 days, hind limb buds were first observed (Stage 32; Fig. 6D). Forelimbs were first noted after 37 days. Metamorphosis completed at 40 days, with emergence of the froglet (Stage 45) around the swamp pool in the first week of July (Fig. 6G).

Eight tadpoles at various stages were collected from a swamp pool in June, 2016. This description is based on two tadpoles in Stage 38 (Fig. 6D; Table 1). We chose Stage 38 because hind limbs are not prominent and tadpole growth is substantial to highlight most key traits. The tadpole is oval and depressed with a brown body and a pale-brown to off-white tail that has translucent fins. The body, tail, and fins have scattered dark brown spots and blotches. The body is also further pigmented with numerous golden iridocytes, which reach sparsely to the tail. The ventral and ventrolateral body sides are translucent, with a few scattered iridocytes. The eyes are black and copper coloured, and of moderate size, positioned dorsolaterally, not visible in ventral view. In lateral view, the body is slightly depressed, and the snout slightly rounded. The heart, gill apparatus, and intestine are visible through the ventral part of the body. The naris is equidistant between the snout and the eye. The spiracle is sinistral, and ventrolaterally positioned at the midbody. The tail fin is moderate, and rounded at the end, while the upper fin is larger than the lower fin. The oral disc is anteroventrally positioned and emarginated laterally. The marginal papillae frame the oral disc across both upper and lower labia, while small submarginal papillae cover the entire inner rim of the oral disc. The upper jaw sheath is narrow and stretched into a wide U-shaped arch and the lower jaw sheath is V-shaped. The labial tooth row formula (LTRF) is 4(2-4)/3(1) (see Fig. 6B). Measurements (mm) of the representative tadpoles (*n* = 2) were as follows: 21.5 total length, 6.8 body length, 14.8 tail length, 3.4 body width, 2.8 body height, 0.9 upper tail fin height, 0.3 lower tail fin height, 2.4 maximum tail length, 0.9 eye-naris distance, 1.2 naris-snout distance, 4.3 snout-spiracle distance, 0.1 internarial distance, 1.6 interorbital distance, and 0.9 eye diameter.

## Discussion

Although the objective of this study was to perform focal-animal samples and instantaneous behavioural scans of *Mercurana myristicapalustris*, we were prevented from making detailed observations on a larger sample of individuals. A primary reason for this difficulty is that the species’ *Myristica* swamp habitat has been reduced from historical extents to just a few fragments (Chandran & Mesta, 2001). The presence of wild elephants rendered most fragments inaccessible for us in the evenings for reasons of security.

During repeated visits to two sites over different years, we noticed yearly variation in periodicity of *Mercurana*’s annual breeding period. One site was our primary study area from 2012 to 2017, and the second was at Sasthanada, where we recorded this species in 2017 and 2018. During this period at these sites, we noted male reproductive activity to range from two-weeks in some years, to barely one-month, between mid-May and early June.

Our observations in this study indicate that *M. myristicapalustris* exhibits a combination of characteristics of both explosive and prolonged breeding strategies, which are two extremes of a spectrum of amphibian breeding systems. Males secured optimal breeding sites by actively defending territories, despite the species’ apparently explosive breeding strategy. Males also engaged in active combat with intruding conspecific males, and sites were occupied by defending males on multiple nights. Male site fidelity to exposed perches has been explained by two hypotheses that state (a) that it provides better visual field that improves visual and acoustic communication and, (b) that it facilitates the defense of territories and search for mates (Abrunhosa & Wogel, 2004; Tsuji, 2004; Wells, 2007). Although sexual dimorphism too has been associated with male–male territorial combat behaviour (Magalhães et al., 2018), sexual dimorphism in *M. myristicapalustris* may not be an evolutionary response to male territoriality.

Male *M. myristicapalustris* have a repertoire of different call types, of which we recorded an advertisement call, an encounter call, and three calls for which the function is as yet unknown. Several species of rhacophorids are known to possess diverse vocal repertoires, producing a large number of distinct calls that are variable in structure, duration, amplitude and frequency, in no predictable order (Rowley et al., 2011). In spite of noting territorial, encounter, fighting and displacement behaviours in *M. myristicapalustris*, it is difficult with our low encounter rates and poor sample size, to categorize our recordings to any of these particular aggressive behaviour categories (Toledo et al., 2015). A study by Zhu et al. (2017) implied that the related male *Kurixalus odontotarsus* added specific notes in compound calls (that contain two kinds of notes) to suppress effects of an opponent male’s calls in male-male competition, while also providing females with information about the male’s ability to attract mates. We are yet unaware of any such suppressor notes in *M. myristicapalustris*, although our study was not detailed enough to discern such a possibility. Many other species within the Rhacophoridae, including those occurring in the Western Ghats, such as species of *Beddomixalus* (Abraham et al., 2013), *Raorchestes* (Bee, Suyesh & Biju, 2013a) and *Pseudophilautus* (Bee, Suyesh & Biju, 2013b) also produce vocal repertoires of varying complexity.

The two females observed in this study chose to mate with males that staked out territories closer to breeding sites in the lowest parts of the swamp that had the potential to be flooded for the longest period of time. Eggs were loosely deposited in shallow burrows made by the female on the swamp floor during the pre-monsoon season, and this can be confirmed as a variant of a terrestrial nest. Utilization of burrows of varying depths as egg deposition and development sites is known primarily in terrestrial and fossorial frogs (Bailey & Roberts, 1981; Kaminsky, Linsenmair & Grafe, 1999; Wells, 2007). Terrestrial eggs have been known to have an advantage over aquatic eggs, especially in their improved embryonic respiratory environment, and thus freeing animals to colonize habitats without permanent water bodies (Touchon & Warkentin, 2008). However, terrestrial eggs experience new risks from desiccation and terrestrial predators (Martin, 1999). So, many terrestrial egg laying species oviposit in terrestrial nests in which the necessary humidity is maintained, such as the moss nests of the Australian moss frog (*Bryobatrachus nimbus*) (Mitchell, 2002). The Australian brown toadlet (*Pseudophryne bibroni*) however, oviposit in a terrestrial nest under moist leaf litter, with tadpoles emerging upon being flooded (Mitchell, 2005), just as observed in *M. myristicapalustris*. It has been noted in terrestrial nests of the red-backed toadlet (*Pseudophryne coriacea*) that hypoxia triggers egg hatching (O’Brien et al., 2018). Our observations on *M. myristicapalustris* showed that in clutches oviposited at the same time, rain-induced inundation of nest sites triggered egg hatching while clutches in sites that were not flooded took longer to hatch. We need more detailed observations to test the role of hypoxia in *M. myristicapalustiris* larval emergence.

Time of oviposition in both instances of breeding that we observed was just before the intensive southwest monsoons, which makes landfall on the southwest coast of India annually around the beginning of June. Our observations in this study provide insight into an error we made in our previous report of amplectic behaviour in this species. We had reported in Abraham et al. (2013) that when the amplectic pair began oviposition in the leaf litter, “both individuals slowly changed colour, becoming almost inconspicuous on the forest floor”. We now know that there is no such colour change, and that the illusion of a different colour in the amplectic pair was caused by poor lighting at the time.

As with the monotypic *M. myristicapalustiris*, closely related taxa such as *Nasutixalus* (three species of phytothelm breeders), *Beddomixalus* (one species that gel nests terrestrially on swamp edges) and *Kurixalus* (15 species, of which some species are known to utilize swamp systems for terrestrial nesting, some others are phytothelm breeders, and breeding mode in the remainder is unknown) are relatively species poor (Frost, 2018; Fig. S1). This pattern is in contrast to *Philautus, Raorchestes*, and *Pseudophilautus*, which are direct developers (Meegaskumbura et al., 2015); these latter genera are species-rich, containing >50 species each (Frost, 2018; Fig. S1). This disparity in species numbers per genus in relation to reproductive strategy may be an artefact of the dominant climatic regime in the region today. As such, we suspect swamp-breeding taxa may be depauperate in the Western Ghats owing to the absence today of an Equatorial, perhumid climatic regime, which had been more dominant in the Indian Peninsula in the past, such as during the Miocene (Kern et al., 2013). Direct-developing clades may now contain larger species numbers due to direct development being more optimal in today’s dominant seasonal climate (Vijayakumar et al., 2016).

The breeding cycles of the majority of life forms of the Western Ghats, including amphibians, seem to be selected by the monsoon system that is the primary harbinger of rains on the Indian Subcontinent (Krishnan, 2002; Van Bocxlaer et al., 2009). Of the three phases of the Indian monsoon (the other two being the pre-monsoon and northeast monsoon), the southwest monsoon is the most influential, bringing the bulk of precipitation over the Indian Subcontinent. However, a few frog species in these mountains have their breeding activity timed with the pre-monsoons, which precede the southwest monsoon (Abraham et al., 2013; Zachariah et al., 2012). Such pre-monsoon breeders often complete their reproductive activities explosively prior to the southwest monsoon, apparently as a strategy to allow early establishment of larvae in rejuvenating aquatic habitats by the time of the monsoons (Abraham et al., 2013; Zachariah et al., 2012). The hybrid reproductive strategy employed by *M. myristicapalustris*, in which they mate explosively in a span of less than a month, and yet maintain specific male territories where females choose mates (characteristic of prolonged breeders), might have to do with the availability of the pre- and southwest-monsoons as a limited climatic window in which to complete development and maturation. Since we have not located *M. myristicapalustris* tadpoles outside of *Myristica* swamps despite repeated surveys, we suspect if the low dissolved oxygen and low pH of the swamp pools of *Myristica* swamp forests, which are stagnant and acidic (Nair et al., 2007), might have a role in influencing the development of the tadpole of this species, and thus the species’ distribution. However, patchily-distributed, relictual *Myristica* swamp forests have been and are still being decimated by anthropogenic factors such as habitat conversion and the effects of climate change (Priti et al., 2016). Furthermore, our study throws some light on larval emergence success of *M. myristicapalustris* and its potential relationship with timely monsoon rains, where any deviation from the norm could impact froglet recruitment into the population. These factors might render *Mercurana* vulnerable to the threat of extinction, given that its reproductive mode restricts it to what is a critically endangered habitat type and a short climatic window. Additional studies on aspects of the species’ biology such as its association with its specific reproductive habitat and ongoing climatic fluxes are urgently required for immediate and future conservation intervention.

## Conclusion

Our observations of *M. myristicapalustiris* in the wild revealed their reproductive activities to be restricted to a brief, few-weeks long reproductive period, during which territorial males attract selective females to breed by terrestrial nesting in a specific type of tropical freshwater swamp ecosystem. Given that this species has been located only in a few fragments of the highly threatened *Myristica* swamp ecosystem of south Kerala, it is pertinent that more detailed exploration and studies are carried out to understand the accurate distribution of its entire metapopulation and its relationship to its habitat. Our results identify some key traits of this highly restricted frog taxon, which will help throw light on the evolution of this clade of Asian tree frogs.

##  Supplemental Information

10.7717/peerj.5934/supp-1 Supplemental Information 1 Type 1 and 2 calls Raw data of frog vocalization.

10.7717/peerj.5934/supp-2 Supplemental Information 2 Type 3 call Raw data of frog vocalization.

10.7717/peerj.5934/supp-3 Supplemental Information 3 Type 4 and 5 calls Raw data of frog vocalization.

10.7717/peerj.5934/supp-4 Figure S1 ML Tree of Philautus-Kurixalus-Mercurana-Raorchestes clade Maximum Likelihood Tree of Philautus-Kurixalus-Mercurana-Raorchestes sub-clade of the larger Rhacophoridae presented in Abraham et al. (2013). This phylogenetic tree shows the relationship of Mercurana with other allied genera within the clade. Also shown in the tree are the number of described species per genus, and breeding modes (Direct-development, Phytothelm (in vegetation cavities) breeding and Terrestrial gel nesting) known of the various taxa in this clade.

10.7717/peerj.5934/supp-5 Figure S2 Annual local precipitation graph Graph showing annual local precipitation during the study period (2015-2017).

10.7717/peerj.5934/supp-6 Supplemental Information 6 Annual local precipitation data Raw data of annual local precipitation (2015-17) acquired from the Kerala Livestock Development Board Bull Station & Farm, Kulathupuzha, which was the closest rain gauge available to us.

## Additional Information and Declarations

### Field Study Permissions

The following information was supplied relating to field study approvals (i.e., approving body and any reference numbers): Fieldwork at the designated site was conducted with permissions and guidelines from the Kerala State Forest Department under the research permit # WL10-38972/2016.

### Data Availability

The following information was supplied regarding data availability: The raw data are provided in the Supplemental Files.
