# Supplementary figures and images for: Reproduction and metamorphosis in the Myristica Swamp tree frog, Mercurana myristicapalustris (Anura: Rhacophoridae)
Source: PeerJ. 2018 Nov 21;6:e5934. doi: 10.7717/peerj.5934 (PMC6252067; doi:10.7717/peerj.5934)

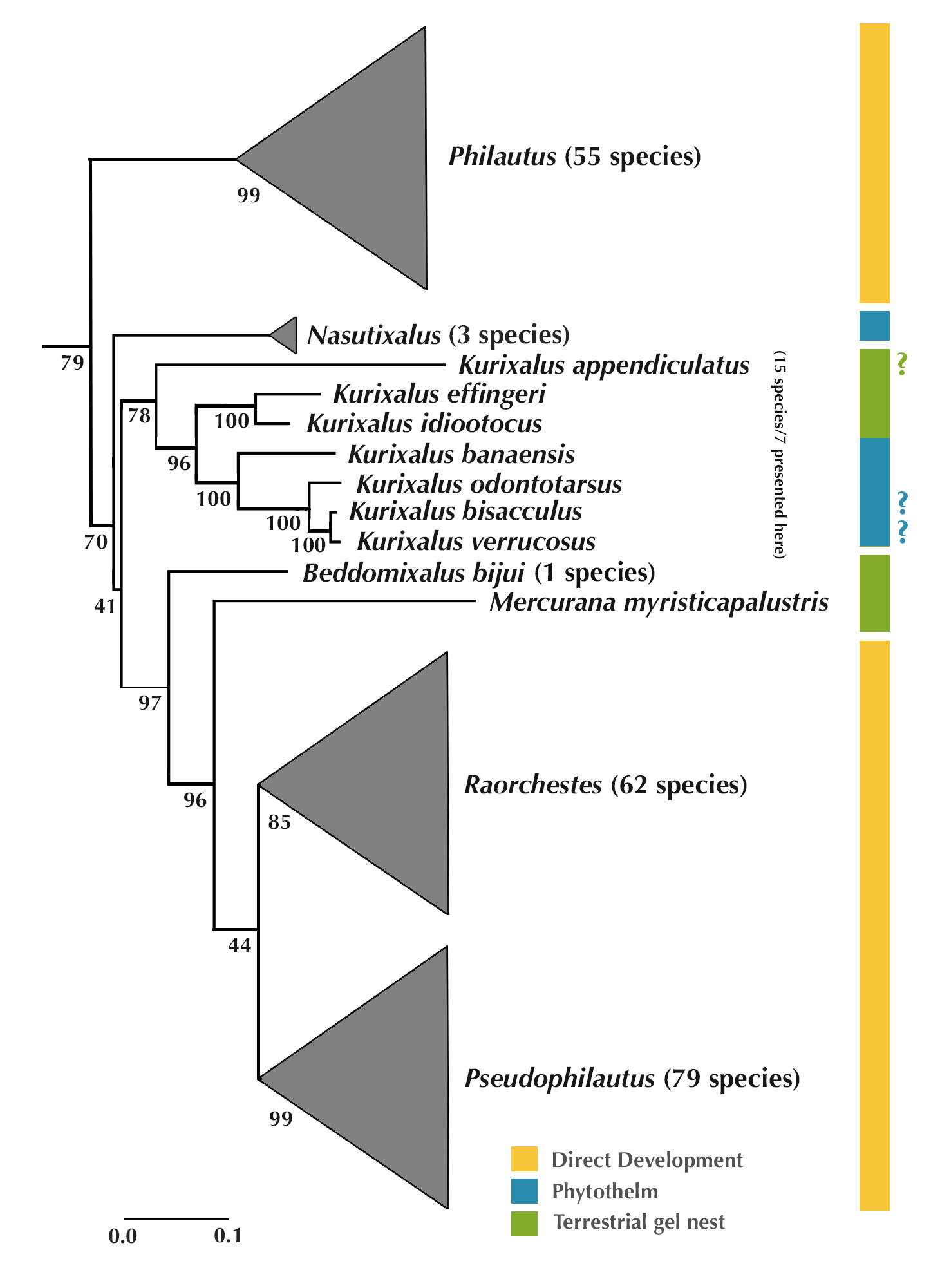

Supplement: Figure S1 — Maximum Likelihood Tree of Philautus-Kurixalus-Mercurana-Raorchestes sub-clade of the larger Rhacophoridae presented in Abraham et al. (2013). This phylogenetic tree shows the relationship of Mercurana with other allied genera within the clade. Also shown in the tree are the number of described species per genus, and breeding modes (Direct-development, Phytothelm (in vegetation cavities) breeding and Terrestrial gel nesting) known of the various taxa in this clade. [file peerj-06-5934-s004.jpg]

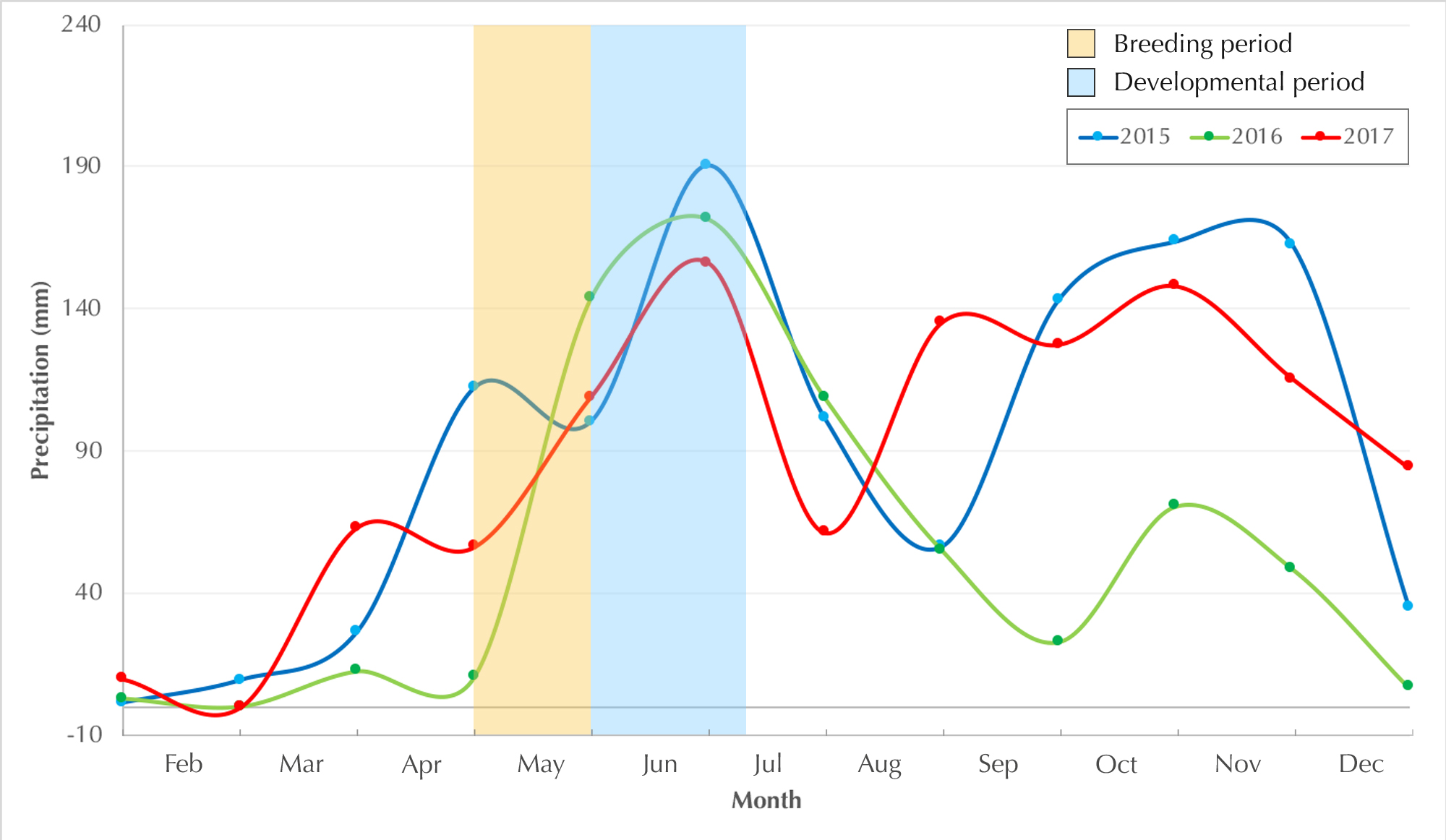

Supplement: Figure S2 — Graph showing annual local precipitation during the study period (2015-2017). [file peerj-06-5934-s005.jpg]
